# “DIAGNOSE AND ADIOS”: MULTIPERSPECTIVE INSIGHTS ON FORMAL SERVICE USE IN DEMENTIA FAMILY CAREGIVERS

**DOI:** 10.1093/geroni/igae098.0162

**Published:** 2024-12-31

**Authors:** Annabelle Greenfield, Hannah Mason, Francesco Osso, Katerina Bumbalova, Rachel Bloom, Francesca Falzarano

**Affiliations:** University of Southern California, San Marcos, California, United States; Weill Cornell Medicine, New York, New York, United States; Fordham University, New York, New York, United States; University of Washington, Seattle, Washington, United States; Fordham University, New York, New York, United States; University of Southern California, Los Angeles, California, United States

## Abstract

Over 11 million unpaid family caregivers currently provide care to 6.7 million individuals with Alzheimer’s disease and related dementias (ADRD). The diverse roles associated with ADRD caregiving range from physical care responsibilities, providing emotional support, in addition to more complex roles such as medical/legal/financial decision-making. Formal and informal supports (e.g., respite, training/education, social support) may be protective against adverse stress-related outcomes such as depression, anxiety, and burden. Despite the existence of such supports, caregivers’ utilization rates remain remarkably low. Using focus group methodology, the current study explored ADRD caregivers’ experiences with the formal services system and identified barriers that hinder service access and utilization. Five semi-structured focus groups were conducted with ADRD family caregivers (n=13) and Dementia Care Community Partners (n=17). Qualitative findings identified three overarching concepts: 1) “Pathways to Preparedness,” categorized primary domains of unmet needs of support, specifically regarding education, emergency preparedness, financial/legal guidance, and mental health. 2) “Multi-Level Barriers” examines obstacles identified across the individual, provider, and systemic levels that hinder service identification and access. 3) “Bridging the Gap” illustrates technology’s potential in mending the broken needs-to-services continuum by providing tailored and personalized support to caregivers. Findings underscore the complex disconnect between caregivers’ needs and existing services, leading to a lack of preparedness for caregiving roles and stress-related adverse outcomes. Yet, a viable solution lies in web-based behavioral interventions, creating a clear pathway for caregivers to identify, access, and utilize formal services, thus improving their overall experiences and outcomes.

